# HIV-1 Tat Dysregulates the Hypothalamic-Pituitary-Adrenal Stress Axis and Potentiates Oxycodone-Mediated Psychomotor and Anxiety-Like Behavior of Male Mice

**DOI:** 10.3390/ijms21218212

**Published:** 2020-11-03

**Authors:** Mohammed F. Salahuddin, Fakhri Mahdi, Jason J. Paris

**Affiliations:** 1 Department of BioMolecular Sciences, School of Pharmacy, University of Mississippi, University, MS 38677-1848, USA; smohamme@go.olemiss.edu (M.F.S.); fmahdi@olemiss.edu (F.M.); 2 Research Institute of Pharmaceutical Sciences, University of Mississippi, University, MS 38677, USA

**Keywords:** adrenal insufficiency, antalarmin, hypothalamic-pituitary-adrenal axis, opioids, trans-activator of transcription, RU-486

## Abstract

Human immunodeficiency virus (HIV) is associated with co-morbid affective and stress-sensitive neuropsychiatric disorders that may be related to dysfunction of the hypothalamic-pituitary-adrenal (HPA) stress axis. The HPA axis is perturbed in up to 46% of HIV patients, but the mechanisms are not known. The neurotoxic HIV-1 regulatory protein, trans-activator of transcription (Tat), may contribute. We hypothesized that HPA dysregulation may contribute to Tat-mediated interactions with oxycodone, a clinically-used opioid often prescribed to HIV patients. In transgenic male mice, Tat expression produced significantly higher basal corticosterone levels with adrenal insufficiency in response to a natural stressor or pharmacological blockade of HPA feedback, recapitulating the clinical phenotype. On acute exposure, HIV-1 Tat interacted with oxycodone to potentiate psychomotor and anxiety like-behavior in an open field and light-dark transition tasks, whereas repeated exposure sensitized stress-related psychomotor behavior and the HPA stress response. Pharmacological blockade of glucocorticoid receptors (GR) partially-restored the stress response and decreased oxycodone-mediated psychomotor behavior in Tat-expressing mice, implicating GR in these effects. Blocking corticotrophin-releasing factor (CRF) receptors reduced anxiety-like behavior in mice that were exposed to oxycodone. Together, these effects support the notion that Tat exposure can dysregulate the HPA axis, potentially raising vulnerability to stress-related substance use and affective disorders.

## 1. Introduction

Human immunodeficiency virus type 1 (HIV-1) continues to be a serious health concern with over 1 million infected individuals in the U.S. [1]. Combined antiretroviral drugs (cART) have greatly increased the life expectancy of people living with HIV and have reduced the incidence of HIV-associated dementias [2]. However, patients still contend with a constellation of neurological symptoms (i.e., neuroHIV), likely due to poor retention of cART within the central nervous system (CNS) and its inability to target neurotoxic HIV-1 proteins and latent viral reservoirs [3]. Moreover, opioid use may exacerbate neuroHIV symptomology, a concern that extends to both illicit and licit opioid users given that a potentially large proportion (8–52%) of HIV-1 infected patients are prescribed opioids [4,5,6].

A common, but under-investigated neurological complication of HIV involves disruption of the hypothalamic-pituitary-adrenal (HPA) stress axis. Despite treatment with cART, 14–46% of HIV+ patients demonstrate a dysregulated HPA axis characterized by elevated basal glucocorticoids with seemingly paradoxical adrenal insufficiency in response to a stressor [7,8,9]. Communication within the HPA axis is mediated by corticotrophin-releasing factor (CRF) secreted from the paraventricular nucleus of the hypothalamus which activates adrenocorticotrophic hormone (ACTH) secretion from the anterior pituitary. Circulating ACTH stimulates adrenal release of glucocorticoids (primarily cortisol in humans and corticosterone in rats and mice) which act at CNS targets, including glucocorticoid receptors (GR), to attenuate the HPA axis response and restore the sympathetic/parasympathetic tone within the CNS. Disruption of either the production of circulating glucocorticoids or glucocorticoid targets is expected to perturb CNS homeostasis in response to a variety of stressors, including physical, psychological, immune, or drug-of-abuse related challenges [10]. HPA axis impairments promote vulnerability to affective and substance use-related disorders [11].

The mechanisms of HIV-mediated HPA dysregulation are not known but may involve neurotoxic HIV-1 proteins. One important therapeutic target that may contribute to these effects is the regulatory protein, trans-activator of transcription (Tat). HIV-1 Tat exerts direct effects on neurons to promote excitotoxic injury and activates monocyte-derived cells (and astrocytes to a lesser extent) to promote neuroinflammation via cytokine production [12]. We have recently observed that transgenic expression of Tat is sufficient to elevate circulating glucocorticoids and central CRF in mice [13]. These effects were augmented by exposure to the clinically-prescribed opioid, oxycodone consistent with preclinical demonstrations of Tat/opioid synergy [13,14,15].

In the present study, we hypothesized that HIV-1 Tat and/or oxycodone would interact to promote HPA axis dysregulation, potentiating oxycodone-mediated effects including psychomotor stimulation and affective dysfunction in a conditionally-inducible Tat transgenic mouse model. We further expected pharmacological blockade of GR and/or CRF receptors to restore HPA function and to ameliorate behavioral deficits.

## 2. Results

### 2.1. Experiment 1: HIV-1 Tat Expression Causes Adrenal Insufficiency and Potentiates Oxycodone’s Psychomotor Effects

**Figure 1:**
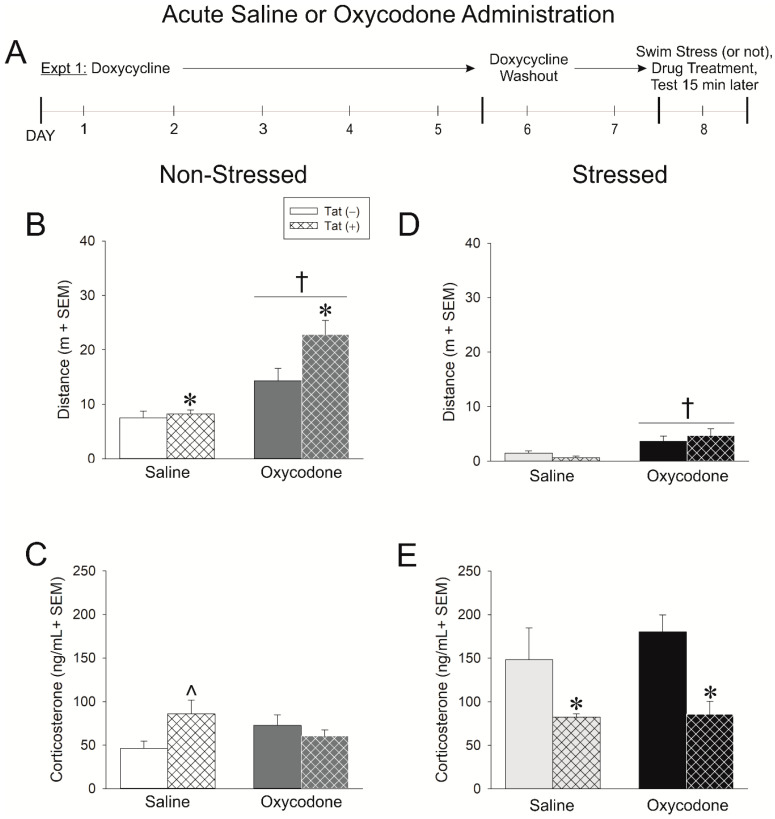
(**A**) In experiment 1, human immunodeficiency virus (HIV)-1 trans-activator of transcription (Tat) expression was induced in Tat(+) males (hatched bars), or not induced in Tat(−) controls (open bars), via administration of doxycycline (30 mg/kg, i.p., once daily for 5 days with 2 days of washout). Mice were either stressed via forced swim for 15 min (panels (**D**,**E**)) or not (panels (**B**,**C**)) and acutely-administered saline or oxycodone (3 mg/kg, i.p.) 15 min prior to assessment in an open field and light-dark transition test (*n* = 8–12/group). (**B**) Distance (m) traveled in an open field and (**C**) circulating corticosterone among non-stressed mice. (**D**) Distance (m) traveled in an open field and (**E**) circulating corticosterone among stressed mice. * indicates a main effect of genotype wherein Tat(+) mice differ from Tat(−) controls. † indicates a main effect for oxycodone to differ from saline-administered mice. ˄ indicates an interaction wherein saline-administered Tat(+) mice differ from respective Tat(−) controls, *p* < 0.05.

In Experiment 1, Tat-tg male mice had HIV-1 Tat expression induced (or not) via doxycycline administration for five days. After two days of doxycycline washout (to limit non-specific anti-inflammatory effects), mice were (or were not) exposed to a 15-min swim stress. Following stress, mice were acutely administered an injection of saline (0.9%, i.p.) or oxycodone (3 mg/kg, i.p.) and psychomotor and anxiety-like behavior were assessed 15 min later (Figure 1A).

The induction of HIV-1 Tat significantly potentiated the psychomotor response to acute oxycodone, increased anxiety-like behavior in a light-dark task, and dysregulated the HPA stress axis. Compared to Tat(−) controls, Tat(+) mice traveled a significantly greater distance [*F*(1,34) = 5.00, *p* < 0.05] (Figure 1B; see *) and velocity [*F*(1,34) = 5.00, *p* < 0.05] (Table 1) in an open field and spent less time engaged in rearing behavior [*F*(1,34) = 5.04, *p* < 0.05] (Table 1). Irrespective of genotype, oxycodone significantly increased the distance [*F*(1,34) = 27.16, *p* < 0.05] (Figure 1A; see †) and speed [*F*(1,34) = 27.34, *p* < 0.05] of travel and decreased the frequency [*F*(1,34) = 5.60, *p* < 0.05] and time spent rearing [*F*(1,34) = 68.97, *p* < 0.05], compared to saline administration (Table 1). In a light-dark transition task, Tat(+) mice spent significantly less time in the brightly-lit compartment [*F*(1,32) = 8.69, *p* < 0.05] and made fewer transitions between compartments [*F*(1,32) = 4.99, *p* < 0.05] compared to Tat(−) controls (Table 1). No significant difference was observed in the latency to transition to the dark compartment (Table 1). When circulating corticosterone was assessed, a significant interaction between genotype and oxycodone administration was revealed [*F*(1,31) = 5.15, *p* < 0.05] (Figure 1C). Tat(+) mice had significantly greater basal corticosterone compared to Tat(−) mice (*p* = 0.02; Figure 1C; see *). Oxycodone increased corticosterone in Tat(−) mice such that this genotype difference was obviated following drug administration (Figure 1C).

Activation of the HPA axis via 15-min swim stress altered the psychomotor, anxiety-like, and glucocorticoid response of mice. Following swim stress, motor behavior in the open field was notably reduced among all groups compared to that previously observed in non-stressed mice. As seen before, oxycodone significantly increased the distance [*F*(1,30) = 13.83, *p* < 0.05] and speed [*F*(1,30) = 13.50, *p* < 0.05] of travel; however, no differences were observed between Tat(−) and Tat(+) mice (Figure 1D), nor were any differences observed in the frequency or time spent rearing (Table 1). Similarly, swim stress attenuated any prior anxiety-like differences observed on the light-dark transition test (Table 1). As expected, circulating corticosterone was greater among Tat(−) mice that underwent swim stress; however, their Tat(+) counterparts mounted a significantly reduced response in comparison [*F*(1,29) = 13.88, *p* < 0.05] (Figure 1E; see *). Together, these data demonstrate that HIV-1 Tat expression in male mice increases basal corticosterone, but produces an adrenal insufficiency upon HPA activation, recapitulating the clinical phenotype reported among HIV+ patients.

### 2.2. Experiment 2: Repeated Exposure to Oxycodone Increases the Tat-Mediated Psychomotor and Glucocorticoid Stress Response

**Figure 2:**
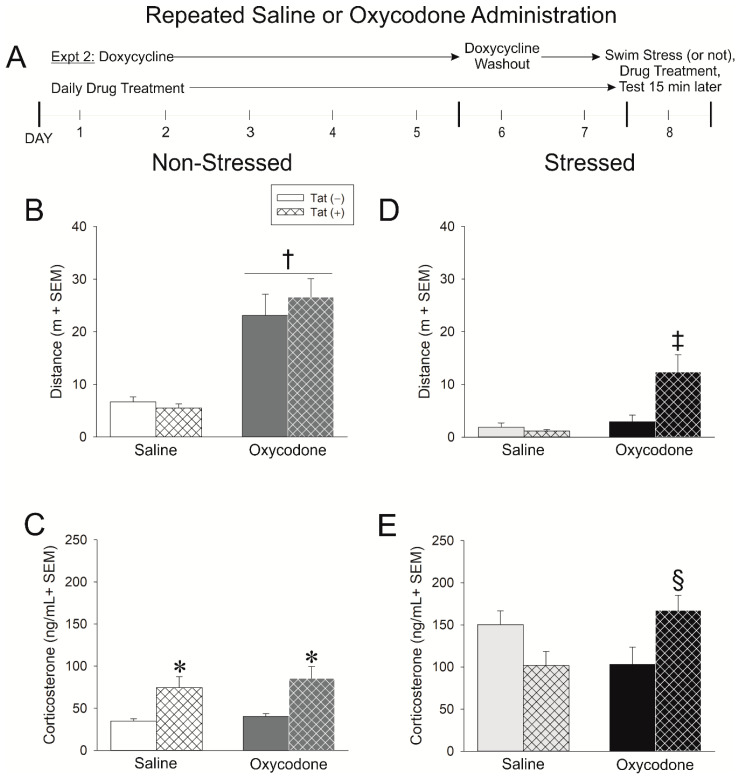
(**A**) In experiment 2, Tat(−) (see open bars) and Tat(+) (see hatched bars) mice were administered saline or oxycodone (3 mg/kg, i.p., once daily for 7 days) concurrent with the induction of HIV-1 Tat via doxycycline (30 mg/kg, i.p., once daily for 5 days with 2 days of doxycycline washout). Mice were stressed via forced swim for 15 min (panels (**D**,**E**)) or not (panels (**B**,**C**)), were administered the last treatment of repeated saline or oxycodone, and 15 min later were assessed in an open field and light dark transition test (*n* = 8–10/group). (**B**) Distance (m) traveled in an open field and (**C**) circulating corticosterone in among non-stressed mice. (**D**) Distance (m) traveled in an open field and (**E**) circulating corticosterone among stressed mice. * indicates a main effect of genotype wherein Tat(+) mice differ from Tat(−) controls. † indicates a main effect for oxycodone to differ from saline-administered mice. ‡ indicates an interaction wherein oxycodone-administered Tat(+) mice differ from all other mice. § indicates an interaction wherein oxycodone-administered Tat(+) mice differ from their respective Tat(−) controls & Tat(+) saline-administered mice, *p* < 0.05.

While the initial opioid response is indicative of later abuse liability, many HIV+ patients are prescribed oxycodone and are exposed repeatedly. How repeated oxycodone exposure modifies the HPA stress response in Tat-exposed mice was of interest. In Experiment 2, Tat-tg male mice had HIV-1 Tat expression induced (or not) via doxycycline administration for five days (with two days of washout). During this time, mice received daily injections of saline (0.9%, i.p.) or oxycodone (3 mg/kg, i.p.). Mice were (or were not) exposed to a 15-min swim stress prior to testing (Figure 2A).

Repeated oxycodone significantly increased the distance [*F*(1,32) = 46.98, *p* < 0.05] (Figure 2B; see †) and velocity [*F*(1,32) = 46.68, *p* < 0.05] (Table 2) of travel among mice while reducing the frequency [*F*(1,32) = 6.08, *p* < 0.05] (Table 2) and time [*F*(1,32) = 11.18, *p* < 0.05] (Table 2) spent rearing, irrespective of their genotype. An interaction was observed for anxiety-like behavior in the light-dark transition test wherein Tat(+) mice demonstrated a significant decrease in the latency to transition to the dark compartment when administered repeated oxycodone [*F*(1,32) = 4.05, *p* = 0.05]; no such effect was observed on Tat(−) controls (Table 2).

Likewise, Tat(+) mice spent significantly less time in the brightly-lit compartment, compared to Tat(−) mice [*F*(1,32) = 3.99, *p* = 0.05] (Table 2). Irrespective of genotype, repeated oxycodone induced more light-dark compartmental transitions than did repeated saline [*F*(1,32) = 4.93, *p* = 0.05] (Table 2). Tat(+) mice demonstrated significantly greater circulating corticosterone than did Tat(−) controls [*F*(1,31) = 14.79, *p* < 0.05] (Figure 2C; see *). When considered in light of the data collected in Experiment 1, repeated oxycodone attenuated HPA axis activation among control mice and commensurately potentiated their psychomotor response to the drug, similar to that of Tat(+) mice. These data demonstrate the effects of repeated opioid exposure on the HPA axis and related behavior.

Repeated oxycodone injection also altered the psychomotor and HPA axis response to a stressor, particularly among Tat(+) mice. As seen in Experiment 1, overall motor behavior was reduced following swim stress compared to that observed in non-stressed mice. Repeated oxycodone and genotype interacted such that oxycodone–administered Tat(+) mice traveled a significantly greater distance [*F*(1,29) = 7.01, *p* < 0.05] (Figure 2D; see ‡) and velocity [*F*(1,29) = 7.10, *p* < 0.05] (Table 2) than did any other group. No differences in rearing frequency or time were observed. Notably, repeated oxycodone decreased the time spent in the brightly-lit compartment of the light-dark transition test [*F*(1,29) = 3.86, *p* = 0.05] (Table 2). No differences were observed in the latency to the dark compartment or the number of transitions between compartments (Table 2). As observed in Experiment 1, swim stress increased circulating corticosterone; however, repeated oxycodone significantly interacted with genotype such that stress-exposed Tat(+) mice demonstrated a greater increase in corticosterone following repeated oxycodone injection [*F*(1,29) = 9.60, *p* < 0.05] (Figure 2E; see §).

### 2.3. Experiment 3: Glucocorticoid and CRF Receptors Are Involved in the Psychomotor, Anxiety-Like, and HPA Axis Response to Oxycodone

**Figure 3:**
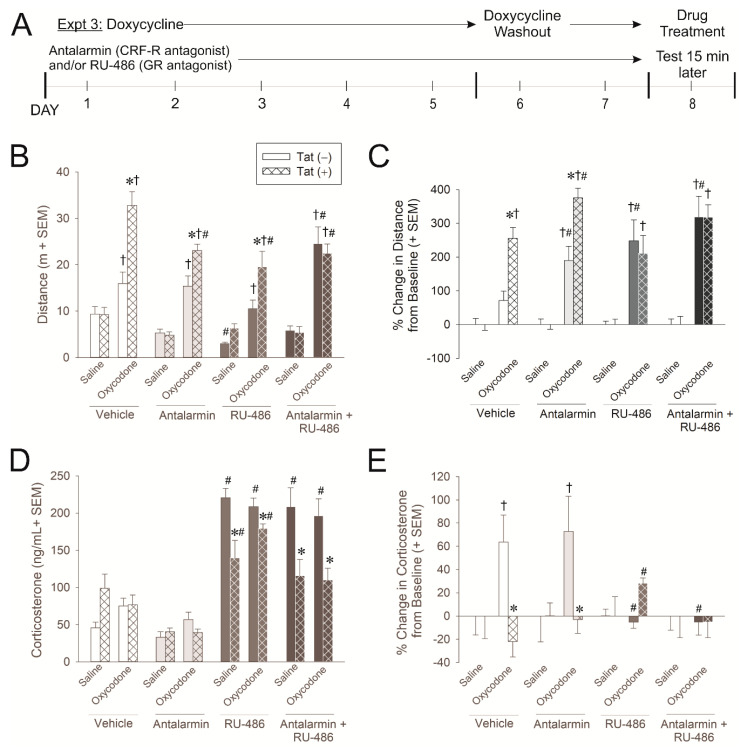
(**A**) In experiment 3, Tat(−) (see open bars) and Tat(+) (see hatched bars) mice were administered antalarmin (corticotrophin-releasing factor receptor antagonist; 20 mg/kg, i.p. for 6 days) and/or RU-486 (glucocorticoid receptor antagonist; i.p., 20 mg/kg, i.p. for 7 days) concurrent with the induction of HIV-1 Tat via doxycycline (30 mg/kg, i.p., once daily for 5 days with 2 days of doxycycline washout). Mice were treated with the final dose of antalarmin and/or RU-486 and then challenged with saline or oxycodone (3 mg/kg, i.p.) and assessed in an open field and light-dark transition test (*n* = 8–10/group). (**B**) Distance (m) traveled in an open field and (**C**) the proportional change from baseline in distance traveled in open field. (**D**) Circulating corticosterone and (**E**) the proportional change from baseline in circulating corticosterone. * indicates an interaction wherein Tat(+) mice differ from respective Tat(−) controls. † indicates an interaction wherein oxycodone-administered mice differ from respective saline-administered controls. # indicates an interaction wherein the denoted group differs from their respective vehicle controls, *p* < 0.05.

In order to begin identifying the important aspects of the HPA axis that are involved in the psychomotor, anxiety-like, and glucocorticoid response to oxycodone and Tat, mice were administered glucocorticoid receptor (GR) and/or corticotrophin-releasing factor-receptor (CRF-R) inhibitors concurrent with doxycycline administration (Figure 3A). To block GR and CRF-R, mice were administered RU-486 (a.k.a. mifepristone) and/or antalarmin, respectively. Mice received an injection of saline (0.9%, i.p.) or oxycodone (3 mg/kg, i.p.) 15 min prior to testing (Figure 3A).

HIV-1 Tat exposure, oxycodone administration, and pharmacological inhibitors significantly interacted to influence psychomotor behavior as assessed by the distance [*F*(3,122) = 2.86, *p* < 0.05] and speed [*F*(3,122) = 3.49, *p* < 0.05] traveled. As before, oxycodone significantly increased the distance (*p* < 0.0001–0.03; Figure 3B; see †) and speed (*p* < 0.0001–0.03; Table 3) traveled among all mice compared to saline administration. Compared to Tat(−) controls, Tat(+) mice exhibited a significant potentiation in oxycodone-induced distances (*p* < 0.0001–0.007; Figure 3B; see *) and speed (*p* < 0.0001–0.006; Table 3) traveled irrespective of pre-treatment with vehicle, antalarmin, or RU-486. However, when Tat(−) mice were treated with both antalarmin and RU-486 (blocking GRs and CRF-Rs), they demonstrated a potentiation of oxycodone-induced psychostimulation that was commensurate to Tat(+) mice (*p* = 0.003; see #). All Tat(+) mice administered a GR and/or CRF-R inhibitor demonstrated a modest, but significant, reduction in the distance (*p* < 0.0001–0.04; Figure 3B; see #) and speed (*p* < 0.0001–0.002; Table 3) of travel. RU-486 notably reduced the distance (*p* = 0.04; Figure 3B; see #) and speed (*p* = 0.04; Table 3) of travel among Tat(−) controls. These data support the notion that GR- and CRF-mediated feedback plays an important role in the acute behavioral response to opioids and the capacity for Tat to potentiate these effects.

To account for differences in baseline psychostimulation that were caused by inhibitors, the proportional change in distance from each group’s baseline was also analyzed (Figure 3C). Oxycodone did not initially increase the distance traveled among Tat(−) controls; however, blocking either GRs and/or CRF-Rs significantly increased the proportional distance traveled (*p* < 0.0001–0.002; Figure 3C; see †), supportive of an inhibitory role for these receptors in this process. Tat(+) mice demonstrated a proportional oxycodone-mediated increase in psychostimulation irrespective of GR and/or CRF-R inhibition (*p* < 0.0001; Figure 3C; see †).

Compared to vehicle-administration, antalarmin and/or RU-486 significantly increased oxycodone-mediated psychostimulation in Tat(−) mice (*p* < 0.0001–0.02; Figure 3C; see #), suggesting that CRF-R and GR signaling are intact and typically inhibitory of this behavior. Among Tat(+) mice, only antalarmin significantly increased oxycodone-mediated psychostimulation (*p* = 0.01; Figure 3C; see #), suggesting that CRF-Rs remain functional; however, the sensitivity or function of GRs may be perturbed.

GR and CRF-R inhibitors significantly interacted with genotype to influence circulating corticosterone concentrations [*F*(3,121) = 11.54, *p* < 0.05]. Pretreatment with RU-486 (alone or in conjunction with antalarmin) produced a significant and large increase in circulating corticosterone among Tat(−) mice (*p* < 0.0001; Figure 3D; see #), presumably by blocking negative feedback within the HPA axis. However, among Tat(+) mice the RU-486-induced increase in corticosterone was present (*p* < 0.0001; Figure 3D; see #), but significantly attenuated compared to that observed in Tat(−) controls (*p* < 0.0001–0.0006; Figure 3D; see *), further supporting a Tat-induced adrenal insufficiency. Antalarmin did not influence corticosterone levels on its own.

The proportional change in corticosterone from baseline was also analyzed in order to account for differences in basal levels (Figure 3E). Tat, oxycodone administration, and pharmacological inhibitors significantly interacted to influence the proportional increase in corticosterone. Among Tat(−) mice, oxycodone proportionally increased circulating corticosterone (*p* = 0.002–0.007; Figure 3E; see †), and this was attenuated by RU-486 (*p* = 0.002–0.003; Figure 3E; see #), but not antalarmin. Tat(+) mice did not generate the proportional increase observed in Tat(−) controls in response to oxycodone (*p* = 0.0002–0.001; Figure 3E; see *) but did generate a modest, but significant increase following RU-486 (*p* = 0.03; Figure 3E; see #).

### 2.4. Experiment 4: The Time-Course of HPA Axis Activation Was Influenced by Oxycodone Exposure

**Figure 4:**
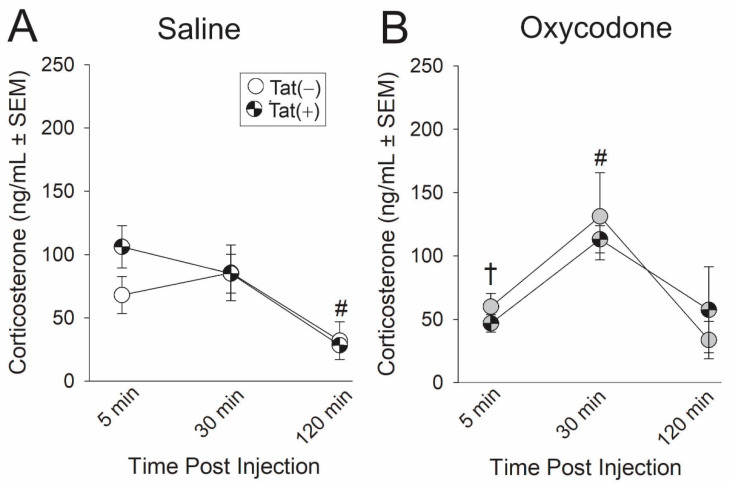
Tat(−) (see open circles) and Tat(+) (see hatched circles) mice (*n* = 5/group) were acutely-administered (**A**) saline (white circles) or (**B**) oxycodone 3 mg/kg, i.p., (gray circles) and circulating corticosterone was measured from serum collected by tail-bleed at 5, 30, and 120 min post-injection. † indicates an interaction wherein oxycodone-administered mice differ from saline-administered mice at t_5_; # indicates an interaction wherein the indicated group differs from their respective t_5_ and t_120_ time-points, *p* < 0.05.

To account for differences in corticosterone levels across different time points, the concentration of circulating corticosterone was measured in plasma from blood samples collected at 5, 30, and 120 min post injection of either saline or oxycodone. Oxycodone administration significantly interacted with the time from injection [*F*(2,32) = 6.11, *p* < 0.05]. Irrespective of genotype, at t_5_ oxycodone-administered mice exhibited significantly lower circulating corticosterone compared to saline-administered mice (*p* = 0.03; Figure 4; see †). At t_30_, oxycodone produced peak plasma corticosterone, significantly differing from t_5_ (*p* = 0.002; Figure 4; see #) and t_120_ (*p* = 0.0006; Figure 4; see #). At t_120_, saline-administered mice demonstrated significantly lower circulating plasma corticosterone as compared to either t_5_ (*p* < 0.0001; Figure 4; see #) or t_30_ (*p* < 0.0001; Figure 4; see #).

## 3. Discussion

The hypotheses that HIV-1 Tat would potentiate oxycodone psychostimulatory and affective behavior concurrent with HPA dysregulation were upheld. Exposure to Tat was sufficient to dysregulate the HPA axis, producing higher basal corticosterone levels with adrenal insufficiency in response to a natural stressor or pharmacological blockade of HPA feedback. These data recapitulate the clinical phenotype of elevated basal cortisol concurrent with secondary adrenal insufficiency which is observed in up to 46% of HIV patients [8]. Given the importance of the HPA response for restoration of CNS homeostasis, dysregulation may raise drug abuse liability and induce vulnerability to affective disorder [11]. Consistent with this, Tat exposure dysregulated the HPA response while potentiating oxycodone’s psychomotor effects and increasing anxiety-like behavior. Given that most patients will be administered oxycodone for multiple days, mice were also administered repeated oxycodone (QD for 7 days). Repeated oxycodone sensitized psychomotor behavior and, when combined with a natural stressor, produced an even greater sensitization in Tat-exposed mice. These findings co-occurred with sensitization of the HPA response supporting the notion that these changes are not independent. Further, pharmacological blockade of GR and/or CRF-R potentiated oxycodone’s psychomotor effects in Tat(−) control mice supporting the involvement of these receptors in opioid-mediated psychostimulation. Blocking GR in Tat(+) mice proportionally increased circulating corticosterone and concurrently reduced oxycodone-mediated psychomotor behavior. Blocking CRF-R ameliorated Tat and oxycodone-mediated effects to promote anxiety-like behavior. These data suggest that the restoration of glucocorticoid activity may be beneficial for Tat/opioid interactions. These data extend findings observed in the clinical HIV+ population and support the notion that Tat contributes to the dysregulation of the HPA stress axis, promoting vulnerability to opioid-sensitization and anxiety-like behavior.

There are well-described and dynamic interactions between the HPA axis and the immune system that likely contribute to the present observations. Expressing Tat in transgenic mice promotes pro- and anti-inflammatory cytokine production in the brain [15,16]. In particular, proinflammatory cytokines may alter the signaling of glucocorticoids at their cognate receptor, GR. Second messengers and transcription factors that regulate cytokine production (including STAT5, p38 MAPK, and NF-κB) are found to inhibit GR signaling [17]. Several reports find nuclear translocation of the GR to be inhibited by STAT5 phosphorylation or by actions of pro-inflammatory cytokines, such as IL-2 or IL-1α, and/or the anti-inflammatory cytokine, IL-4 [18,19,20]. As such, actions of these cytokines may promote GR insensitivity and, thereby, a glucocorticoid-resistant state. Conversely, activation of the HPA axis also exerts modulatory effects on immune signaling. Glucocorticoids promote phagocytic activation of monocyte-derived macrophages and largely suppress proinflammatory cytokine release [21]. Glucocorticoids promote a Th2 profile, suppressing Th1 and Th17 polarization [22,23], and may further promote differentiation to regulatory T cells that suppress immune function [24]. As such, monocyte-derived cells within the brain present as both a target of glucocorticoid actions and a source of potential GR-mediating cytokines. *In lieu* of this dynamic relationship, it is perhaps not surprising that HIV-1 proteins may contribute to the dysregulation of the HPA axis; however, the mechanisms remain to be understood.

Hypercortisolemia is often reported in HIV+ patients and the present data demonstrate that expression of Tat protein is sufficient to produce elevated basal corticosterone in mice. There are several mechanisms that may underlie these effects. In patients, an enzymatic shift towards the production of glucocorticoids has been observed [25]. The cortisol-to-dehydroepiandrosterone (DHEA) ratio is increased in HIV+ patients [26,27] and has been suggested to be a prognostic biomarker for poorer HIV outcomes [28]. Additionally, a basal increase in circulating corticosterone may involve a reduction in glucocorticoid bioavailability. In acquired immunodeficiency syndrome (AIDS), disease progression was associated with increased circulating cortisol binding globulin which would be expected to promote a compensatory increase in adrenal glucocorticoids [29]. Greater basal glucocorticoid concentrations may also be due to a Tat-mediated insensitivity at the GR. In AIDS patients with hypercortisolemia, increased GR density and reduced GR affinity were reported [30]. Decreased GR or CRF-R expression has also been observed [31,32]. In rodents, Tat increases IL-2 and IL-4 in the brain [15,16], and these mediators can induce p38 MAPK, phosphorylating the GR and reducing ligand-binding affinity [23,33]. In support, we have recently found Tat to produce corticosterone insensitivity in murine cultured splenocytes [34]. Lastly, basal glucocorticoids may also be increased by negative regulation of the GR. The GRβ isoform, a dominant negative inhibitor of the bioactive GRα, resides in the nucleus typically at a much lower concentration than GRα [35]. However, proinflammatory cytokines can increase the GRβ:GRα ratio, thereby inactivating GRα [36,37]. Moreover, negative GR regulators, such as Fkbp5, may decrease expression of the GR (which has been observed in HIV+ women) [38]. Thus, there are several mechanisms by which Tat may promote a glucocorticoid resistant state, thereby elevating basal corticosterone levels commensurate to that observed in the HIV+ population.

Concurrent with increased basal corticosterone, Tat promoted adrenal insufficiency when exposed to a natural or pharmacological HPA challenge. While seemingly paradoxical, similar observations of hypercortisolemia and adrenal insufficiency are reported among HIV+ patients [7,25]. The mechanisms are not known but have been suggested to involve a depletion of the “adrenal reserve”. HIV-1 Tat may mediate these effects in part by its capacity to perturb steroidogenesis. All steroidogenesis is dependent on bioavailable cholesterol, the metabolism of which is dysregulated in HIV [39]. Tat protein is sufficient to dysregulate gene expression associated with lipid and cholesterol trafficking and homeostasis [40,41] and increases the production of ceramides that inhibit steroidogenic enzymes [42]. Moreover, Tat exerts toxic effects on mitochondria, the rate-limiting organelle for all steroidogenesis. Tat disrupts oxidative phosphorylation, driving the production of reactive oxygen species, depolarizing mitochondrial membranes, and promoting mitochondrial swelling and the translocation of pro-apoptotic factors [43]. We have found Tat expression in mice to decrease brain concentrations of the glucocorticoid precursor, deoxycorticosterone [34]. Thus, despite increasing basal glucocorticoids, Tat facilitates adrenal insufficiency perhaps via the impairment of steroidogenesis.

HPA dysregulation may confer vulnerability to addiction-related and affective disorders [11]. We and others find Tat to potentiate the sensitizing [13,44] and rewarding effects of drugs of abuse [15] and some studies indicate that HIV-tg rats self-administer psychostimulants to a greater degree [45,46]; albeit, these observations are not uniformly observed [47,48,49]. Preservation of the HPA stress axis may reduce addiction liability. In support, there are many demonstrations of acute stressors reducing conditioned place preference to drugs of abuse, whereas chronic stressors potentiate these effects [50]. Tat’s capacity to dysregulate the HPA axis may contribute vulnerability to the sensitizing and rewarding effects of drugs-of-abuse and these may co-occur with affective disorders. In HIV+ people, higher cortisol-to-DHEA ratios predict higher scores of stressful life events, perceived stress, anxiety and depression symptomology [26,27]. Likewise, the expression of Tat in rodents increases anxiety- and depression-like behavior [51]. Given the ubiquity of the HPA axis in central and peripheral homeostasis, ameliorating Tat-mediated HPA dysregulation may improve outcomes across a multitude of physiological and behavioral parameters.

While the present data demonstrate the sufficiency for Tat to produce HPA dysregulation, additional HIV-1 proteins may exert similar effects, potentially via shared mechanisms. In particular, glycoprotein 120 is a known activator of the HPA axis that increases corticosterone and CRF [52,53]. As well, viral protein R (VPR) can serve as a GR co-activator in human cells and cell lines [54,55]. Moreover, VPR can potentiate HIV-1 Tat effects on viral transcription, perhaps via shared mechanisms as those utilized in GR co-activation [56]. These proteins may thus act alone or in concert to promote HPA dysfunction. Future investigations across HIV-1 models that assess the over-expression or perturbation of glucocorticoid targets may further reveal the important interactions identified by the present pharmacological studies.

Together, the present data illustrate the capacity for HIV-1 Tat to cause GR insensitivity (elevating basal circulating corticosterone levels) and promoting adrenal insufficiency in response to HPA activation to a natural or pharmacological challenge. HPA dysregulation occurred concurrent with anxiety-like behavior and combined Tat and oxycodone-mediated psychostimulation. Repeated oxycodone exposure was able to sensitize the Tat-suppressed HPA response, suggesting potential for pharmacological therapeutic intervention. Blocking GR attenuated combined Tat- and oxycodone-mediated psychostimulation and produced a proportional increase in corticosterone, indicating its importance in these effects. Thus, therapeutics with the capacity to restore the HPA axis may be important mediators of HIV-1 Tat/opioid interactions.

## 4. Materials and Methods

Procedures were preapproved by the Institutional Animal Care and Use Committee (IACUC) at the University of Mississippi (#18-004; approved October, 2017). All experiments were carried in accordance with ethical guidelines defined by the National Institutes of Health (NIH Publication No. 85-23).

### 4.1. Subjects and Housing

Male, adult, transgenic mice (*n* = 324) were bred in the vivarium at the University of Mississippi (University, MS, USA). Mice were housed 2–5 per cage and be kept in a temperature-and humidity-controlled environment on a 12:12 h light:dark cycle (lights off at 09:00 h) with ad libitum access to food and water. HIV-1 Tat_1–86_ is conditionally expressed by administration of doxycycline as described previously [57]. Briefly, Tat(+) mice express a *tat* transgene which is driven by a Glial fibrillary acidic protein-regulated, Tet-on promoter that becomes transcriptionally-active in presence of a doxycycline-sensitive reverse tetracycline-controlled transactivator (rtTA) transcription factor. Tat(−) counterparts express the rtTA transcription factor but lack the tat transgene.

### 4.2. Chemicals

Doxycycline hyclate was prepared fresh daily and dissolved in sterile saline (0.9%) to concentration (30 mg/kg, i.p.; Cayman Chemical, Ann Arbor, MI, USA) per prior methods [13,34]. Oxycodone HCl was dissolved in sterile saline (0.9%) to concentration (3 mg/kg, i.p.; Sigma-Aldrich, St. Louis, MO, USA). We have previously identified oxycodone (3 mg/kg) as an optimal dose to elicit acute psychomotor and behavioral responses [13]. Antalarmin was dissolved in 30% Solutol HS 15 (in saline) to concentration (20 mg/kg, i.p.; Cayman Chemical). Dosing was chosen based on prior demonstrations of CRF-R antagonism [58,59]. RU-486 was suspended in a solution of 30% Solutol HS 15 and 1% DMSO (in saline with 3 drops of Tween 20) to concentration (20 mg/kg, i.p.; Cayman Chemical). Dosing was chosen based on prior demonstrations of GR antagonism [60,61].

### 4.3. Procedure

The expression of HIV-1 Tat was induced in transgenic mice [Tat(+) or Tat(−)] via the administration of doxycycline QD for 5 days i.p. Given that doxycycline can attenuate neuroinflammation and could therefore mask potential effects of Tat, 2 days of doxycycline washout was carried out [62]. All behavioral testing was performed on the 8th day of the protocol (see timelines in panel A of Figure 1, Figure 2 and Figure 3). We have confirmed the presence of Tat protein in the brain of HIV-1 Tat-tg mice [63] and others have reported elevated Tat mRNA in the brain and spinal cord for at least 3 weeks post Tat-induction via doxycyline [64].

#### 4.3.1. Experiment 1: Assessment of Acute Oxycodone Exposure in Non-Stressed and Stressed Mice

To begin to determine the HPA-axis interactions involved in exposure to HIV-1 Tat and acute oxycodone, mice were randomly-assigned to undergo a 15-min swim stress (or not) followed by administration of vehicle (saline, 0.9%, i.p.) or oxycodone (3 mg/kg, i.p.) only once prior to behavioral testing. Fifteen minutes after drug administration, mice were assessed in an open field to determine their psychomotor response followed immediately by assessment in a light-dark transition test to determine anxiety-like behavior (Figure 1A). Across dependent measures for open field, light-dark transition test, and circulating corticosterone, the following non-stressed groups were yielded: Tat(−)/saline (*n* = 8–9), Tat(+)/saline (*n* = 7–8), Tat(−)/oxycodone (*n* = 12), Tat(+)/oxycodone (*n* = 8–9), and the following swim-stressed groups were yielded: Tat(−)/saline (*n* = 8), Tat(+)/saline (*n* = 9), Tat(−)/oxycodone (*n* = 8), Tat(+) oxycodone (*n* = 9).

#### 4.3.2. Experiment 2: Assessment of Repeated Oxycodone Exposure in Non-Stressed and Stressed Mice

Given that most patients are exposed to opioids on a repeated dosing schedule, some mice were administered sterile saline (0.9%) or oxycodone (3 mg/kg) daily throughout the 7-day doxycycline-induction/washout schedule (Figure 2A). As before, mice were randomly-assigned to undergo a 15-min swim stress (or not) followed by an injection of saline (0.9%, i.p.) or oxycodone (3 mg/kg) 15 min prior to behavioral testing (Figure 2A). As in Experiment 1, mice were assessed for psychomotor and anxiety-like behavior. Across dependent measures for open field, light-dark transition test, and circulating corticosterone, the following non-stressed groups were yielded: Tat(−)/saline (*n* = 7–8), Tat(+)/saline (*n* = 10), Tat(−)/oxycodone (*n* = 8), Tat(+)/oxycodone (*n* = 10), and the following swim-stressed groups were yielded: Tat(−)/saline (*n* = 8), Tat(+)/saline (*n* = 8), Tat(−)/oxycodone (*n* = 8), Tat(+) oxycodone (*n* = 9).

#### 4.3.3. Experiment 3: Assessment of Acute Oxycodone Exposure Following GR and/or CRF-R Blockade

To begin to identify the important receptor sites involved in HIV-1 Tat- or oxycodone-mediated disruption of the HPA axis, some mice were pretreated with the GR antagonist, RU-486, and/or the CRF-R antagonist, antalarmin, prior to testing. RU-486 was administered daily throughout the 7-day doxycycline-induction/washout schedule and 30 min prior to behavioral testing (Figure 3A). Antalarmin was administered daily for 6-days during the doxycycline-induction/washout schedule (Figure 3A) and 30 min prior to behavioral testing. All mice received saline or oxycodone (3 mg/kg, i.p.) 15 min prior to behavioral testing (Figure 3A). As in Experiments 1 and 2, mice were assessed for psychomotor and anxiety-like behavior. Across dependent measures for open field, light-dark transition test, and circulating corticosterone, the following groups were yielded: Tat(−)/saline/vehicle (*n* = 8), Tat(+)/saline/vehicle (*n* = 7–8), Tat(−)/oxycodone/vehicle (*n* = 8–9), Tat(+)/oxycodone/vehicle (*n* = 9), Tat(−)/saline/antalarmin (*n* = 8), Tat(+)/saline/antalarmin (*n* = 8–9), Tat(−)/oxycodone/antalarmin (*n* = 8–9), Tat(+)/oxycodone/antalarmin (*n* = 10), Tat(−)/saline/RU-486 (*n* = 8–9), Tat(+)/saline/RU-486 (*n* = 7–8), Tat(−)/oxycodone/RU-486 (*n* = 8–9), Tat(+)/oxycodone/RU-486 (*n* = 7–8), Tat(−)/saline/antalarmin+RU-486 (*n* = 9), Tat(+)/saline/antalarmin+RU-486 (*n* = 8), Tat(−)/oxycodone/antalarmin+RU-486 (*n* = 9–10), Tat(+)/oxycodone/antalarmin+RU-486 (*n* = 9–10).

#### 4.3.4. Experiment 4: Determination of Peak HPA Activation Following Tat or Oxycodone Exposure

A subset of Tat(−) and Tat(+) mice were administered saline (0.9%, i.p.) or oxycodone (3 mg/kg) and had tail-blood collected 5, 30, and 120 min later (*n* = 5/group) in order to assess the time-course for HPA activation. Prior work revealed peak HPA circulating corticosterone levels in HIV-1 Tat females within 30 min of testing [13].

### 4.4. Behavioral Assessment

Mice were tested in a behavioral battery consisting of assessment in an open field followed by a light-dark transition test. All mice were transferred to the behavioral testing room equipped with white noise (70 dB) 30 min prior to testing and were assessed ~2–3 h into the dark phase of the light-dark cycle. Behavior was tracked and encoded by an ANY-maze behavioral tracking system (ver. 5, Stoelting Co., Wood Dale, IL, USA).

#### 4.4.1. Forced Swim Stress Stimulus

The Porsolt forced swim test was used as previously described to activate the HPA stress axis [65]. In brief, mice were placed in room temperature water (~22 °C) and allowed to swim for 15 min. Following swimming, mice were dried with paper towels and returned to their home cages.

#### 4.4.2. Open Field

The open field test was used to assess psychomotor behavior as previously described [13]. In brief, mice were placed in one of the corner of a square Plexiglas^®^ box (40 × 40 × 35 cm; Stoelting Co., Wood Dale, IL, USA) with a brightly-illuminated center (inner 20 cm) and behavior was tracked for 5 min. The total distance traveled (m) and mean velocity (m/s) were used as indices of horizontal motor behavior with rearing assessed as an index of vertical motor behavior.

#### 4.4.3. Light-Dark Transition Test

The light-dark transition test was used to assess anxiety-like behavior as previously described [66]. In brief, mice were placed in the brightly-lit corner of a square Plexiglas box (40 × 40 × 35 cm; Stoelting Co., Wood Dale, IL, USA) that was evenly divided into two compartments (one brightly-lit side and one enclosed dark side) and allowed to explore for 5 min. The latency to enter the dark compartment and the time spent in light chamber was considered an index of anxiety-like behavior. The number of transitions between compartments was used as an index of motor activity.

### 4.5. Corticosterone Assay

#### 4.5.1. Tissue Collection

Immediately following the completion of behavioral testing in Experiments 1–3, mice were sacrificed via cervical dislocation followed by rapid decapitation. Trunk blood was collected, centrifuged at 13,500× *g* at 4 °C for 20 min, and serum was stored at −80 °C. In Experiment 4, tail-blood (~20–50 µL) was collected from mice at 5, 30, and 120 min post-treatment via tail-knick. Serum was collected and stored as described above.

#### 4.5.2. Steroid Extraction

Circulating steroids were isolated from serum using an ether extraction protocol as previously described [13]. Briefly, serum samples were incubated with 1 mL of ice-cold anhydrous ethyl ether and snap frozen in a mixture of acetone and dry ice as described [13]. Supernatant was collected and evaporated to dryness in a fume hood overnight followed by reconstitution to 50× the original volume in extraction buffer. Enzyme-linked immunosorbent assay was performed according to manufacturer instructions (Neogen Life Sciences, Lexington, KY, USA).

#### 4.5.3. Enzyme-Linked Immunosorbant Assay (ELISA)

Circulating corticosterone was assessed via ELISA per manufacturer instructions (Neogen Life Sciences; #402810) and as previously described [13]. For all assays, absorbance was read at 650 nm using a CLARIOstar microplate reader (BMG Labtech Inc., Cary, NC, USA). The antibody cross-reactivity with corticosterone is reported to be 100%. The corticosterone antibody also exerts cross-reactivity with deoxycorticosterone (38%), 6-hydroxycorticosterone (19%), and P4 (5.1%) with minimal cross-reactivity to additional steroids (<2.7%). Respective intra- and inter-assay variances were: 7.5% and 26.8%.

### 4.6. Statistical Analyses

For Experiments 1 and 2, all behavioral and steroid analyses were assessed via separate two-way analyses of variance (ANOVA) with drug condition (i.e., saline or oxycodone) and genotype [i.e., Tat(–) or Tat(+)] as the between-subjects factors. For Experiment 3, behavioral and steroid analyses were assessed via separate three-way ANOVAs with pretreatment (i.e., vehicle, antalarmin, RU-486, or combined antalarmin/RU-486), drug condition (i.e., saline or oxycodone), and genotype [i.e., Tat(−) or Tat(+)] as the between-subjects. For Experiment 4, corticosterone steroid analyses were assessed via repeated measures ANOVA with drug condition (i.e., saline or oxycodone) and genotype [i.e., Tat(−) or Tat(+)] as the between-subjects factors and time (i.e., 5, 30, and 120 min) as the within-subjects factor. Main effects were followed by Fisher’s Protected Least Significant Difference *post hoc* tests in order to determine group differences. Interactions were delineated via simple main effects and main effect contrasts with α controlled for family-wise error. Significant outliers were determined via two-tailed Dixon’s test (no more than 1 outlier/group), which explains any variance in the *n*/group across dependent measures. All analyses were considered significant when *p* ≤ 0.05.

## Figures and Tables

**Table 1:** Motor measures acquired in an open field and anxiety-like/motor measures acquired in a light-dark transition task from Tat(−) and Tat(+) males that were exposed (or not) to a 15-min forced swim stress prior to administration of saline or oxycodone. * indicates a main effect of genotype wherein Tat(+) mice differ from Tat(−) controls. † indicates a main effect for oxycodone to differ from saline administration, *p* ≤ 0.05.

Behavioral Measure	Non-Stressed				Stressed			
	Saline (0.9% *w/v*)		Oxycodone (3 mg/kg)		Saline (0.9% *w/v*)		Oxycodone (3 mg/kg)	
	Tat(−) (*n* = 8–9)	Tat(+) (*n* = 7–8)	Tat(−) (*n* = 12)	Tat(+) (*n* = 8–9)	Tat(−) (*n* = 8)	Tat(+) (*n* = 9)	Tat(−) (*n* = 8)	Tat(+) (*n* = 9)
Mean Velocity (m/s)	0.025 ± 0.004	0.027 ± 0.002 *	0.048 ± 0.008 ^†^	0.076 ± 0.009 ^†^*	0.005 ± 0.001	0.002 ± 0.001	0.012 ± 0.003 ^†^	0.015 ± 0.004 ^†^
Rearing number	39.4 ± 6.2	23.8 ± 3.2	18.1 ± 11.4 ^†^	6.8 ± 2.2 ^†^	4.5 ± 2.4	0.9 ± 0.7	0.8 ± 0.3	0.7 ± 0.2
Rearing Time (s)	31.13 ± 4.74	20.31 ± 2.58 *	3.66 ± 1.56 ^†^	2.14 ± 0.92 ^†^*	2.61 ± 1.53	0.63 ± 0.60	0.29 ± 0.15	0.19 ± 0.09
Latency to first enter dark (s)	61 ± 32	28 ± 19	14 ± 4	22 ± 9	77 ± 45	89 ± 36	63 ± 38	37 ± 33
Light zone time (s)	106 ± 32	17 ± 5 *	75 ± 17	43 ± 9 *	102 ± 40	121 ± 27	112 ± 32	30 ± 5
Number of transitions	9 ± 3	5 ± 1 *	13 ± 2	8 ± 1 *	9 ± 3	7 ± 1	13 ± 3	11 ± 3

**Table 2:** Motor measures acquired in an open field and anxiety-like/motor measures acquired in a light-dark transition task from Tat(−) and Tat(+) males that were exposed (or not) to a 15-min forced swim stress with repeated administration of saline or oxycodone. * indicates a main effect of genotype wherein Tat(+) mice differ from Tat(−) controls. † indicates a main effect for oxycodone to differ from saline-administered mice. ‡ indicates an interaction wherein the denoted group significantly differs from Tat(+), saline-administered controls. § indicates an interaction wherein the denoted group significantly differs from all other groups, *p* ≤ 0.05.

Behavioral Measure	Non-Stressed				Stressed			
	Saline (0.9% *w/v*)		Oxycodone (3 mg/kg)		Saline (0.9% *w/v*)		Oxycodone (3 mg/kg)	
	Tat(−) (*n* = 8)	Tat(+) (*n* = 10)	Tat(−) (*n* = 8)	Tat(+) (*n* = 10)	Tat(−) (*n* = 8)	Tat(+) (*n* = 8)	Tat(−) (*n* = 8)	Tat(+) (*n* = 9)
Mean Velocity (m/s)	0.022 ± 0.003	0.018 ± 0.003	0.077 ± 0.013 ^†^	0.089 ± 0.012 ^†^	0.007 ± 0.003	0.004 ± 0.001	0.01 ± 0.004	0.041 ± 0.011 ^§^
Rearing number	34.8 ± 6.6	25.3 ± 6.2	13.8 ± 4.0 ^†^	19.7 ± 4.1 ^†^	1.1 ± 0.6	1.6 ± 0.5	1.4 ± 0.9	2.0 ± 1.2
Rearing Time (s)	23.3 ± 5.6	19.5 ± 5.5	5.2 ± 1.8 ^†^	9.2 ± 2.3 ^†^	0.4 ± 0.3	0.6 ± 0.2	0.6 ± 0.5	1.0 ± 0.6
Latency to first enter dark (s)	30 ± 12	81 ± 32	38 ± 17	8 ± 2 ^‡^	44 ± 37	38 ± 19	17 ± 9	14 ± 5
Light zone time (s)	116 ± 32	91 ± 25 *	119 ± 25	46 ± 12 *	102± 38	68 ± 19	53 ± 12 ^†^	33± 7 ^†^
Number of transitions	10 ± 2	9 ± 2	17 ± 3 ^†^	11 ± 1 ^†^	10 ± 2	11± 2	15 ± 3	11 ± 2

**Table 3:** Motor measures acquired in an open field and anxiety-like/motor measures acquired in a light-dark transition task from Tat(−) and Tat(+) males that were pretreated with the corticotrophin-releasing factor receptor antagonist, antalarmin, and/or the glucocorticoid receptor antagonist, RU-486, prior to administration of saline or oxycodone. * indicates an interaction wherein Tat(+) mice differ from respective Tat(−) controls. † indicates an interaction wherein oxycodone-administered mice differ from respective saline-administered controls. # indicates an interaction wherein the denoted group differs from their respective vehicle controls, *p* < 0.05.

**Behavioral Measure**	**Vehicle**				**Antalarmin**			
	**Saline (0.9% *w/v*)**		**Oxycodone (3 mg/kg)**		**Saline (0.9% *w/v*)**		**Oxycodone (3 mg/kg)**	
	**Tat(−) (*n* = 8)**	**Tat(+) (*n* = 7–8)**	**Tat(−) (*n* = 8–9)**	**Tat(+) (*n* = 9)**	**Tat(−) (*n* = 8)**	**Tat(+) (*n* = 8–9)**	**Tat(−) (*n* = 8–9)**	**Tat(+) (*n* = 10)**
Mean Velocity (m/s)	0.031 ± 0.006	0.031 ± 0.005	0.053 ± 0.008 ^†^	0.11 ± 0.01 ^†^*	0.018 ± 0.003	0.016 ± 0.002	0.051 ± 0.007 ^†^	0.077 ± 0.005 ^†^*^#^
Rearing number	34 ± 5	32 ± 8	8 ± 4	38 ± 12	16 ± 3	21 ± 6	10 ± 4	12 ± 5
Rearing Time (s)	27.3 ± 4.5	27.7 ± 7.5	1.9 ± 0.7 ^†^	8.6 ± 2.0 ^†^	11 ± 2.4 ^#^	13.6 ± 4.5 ^#^	6.6 ± 3.3 ^†^	2.7 ± 0.9 ^†^
Latency to first entry to dark zone (s)	40.7 ± 14.2	5.3 ± 1.8	10.7 ± 4.9	7.3 ± 1.7	86.9 ± 43.3	125.5 ± 36.4	7.0 ± 1.3 ^†^	6.8 ± 1.9 ^†^
Light zone time (s)	177 ± 31	38 ± 11 *	32. ± 7 ^†^	32 ± 5	104 ± 40	148 ± 34 ^#^	98 ± 31	74 ± 26 ^†^
Number of transitions	14.9 ± 3.6	11.4 ± 2.9	8.4 ± 1.2	9.1 ± 1.3	6.0 ± 2.5	6.1 ± 1.3	14.2 ± 3.5	11.8 ± 2.3
	**RU-486**				**Antalarmin + RU-486**			
	**Saline (0.9% *w/v*)**		**Oxycodone (3 mg/kg)**		**Saline (0.9% *w/v*)**		**Oxycodone (3 mg/kg)**	
	**Tat(−) (*n* = 8–9)**	**Tat(+) (*n* = 7–8)**	**Tat(−) (*n* = 8–9)**	**Tat(+) (*n* = 7–8)**	**Tat(−) (*n* = 9)**	**Tat(+) (*n* = 8)**	**Tat(−) (*n* = 9–10)**	**Tat(+) (*n* = 9–10)**
Mean Velocity (m/s)	0.01 ± 0.001 ^#^	0.021 ± 0.003	0.035 ± 0.006 ^†^	0.065 ± 0.011 ^†^*^#^	0.020 ± 0.003	0.018 ± 0.004	0.082 ± 0.012 ^†#^	0.075 ± 0.007 ^†#^
Rearing number	9 ± 2	17 ± 4	25 ± 20	15 ± 7	12 ± 3	16 ± 5	57 ± 29	11 ± 4
Rearing Time (s)	7.8 ± 2.1 ^#^	13.8 ± 3.5 ^#^	2.9 ± 1.9 ^†^	4.0 ± 1.2 ^†^	8.6 ± 1.9 ^#^	11.0 ± 4.0 ^#^	9.1 ± 2.9 ^†^	1.8 ± 0.4 ^†^
Latency to first entry to dark zone (s)	78.0 ± 35.5	97.3 ± 52.4	16.1 ± 4.5	3.2 ± 0.7	110.0 ± 47.9	23.9 ± 6.4	14.8 ± 4.0 ^†^	3.8 ± 0.9 ^†^
Light zone time (s)	120 ± 43	107 ± 43	116 ± 378 ^#^	8 ± 1 ^†^*	169 ± 40	34 ± 5 *	200 ± 17 ^#^	61 ± 13 *
Number of transitions	3.4 ± 0.7 ^#^	3.1 ± 0.9 ^#^	9.0 ± 2.1	5.0 ± 0.9	7.4 ± 2.4	4.0 ± 0.5	27.4 ± 6.0 ^†#^	33.2 ± 15.5 ^†#^

## References

[1] Centers for Disease Control and Prevention (2018). HIV Surveillance Report (Updated).

[2] Saylor D., Dickens A.M., Sacktor N., Haughey N., Slusher B., Pletnikov M., Mankowski J.L., Brown A., Volsky D.J., McArthur J.C. (2016). HIV-associated neurocognitive disorder--pathogenesis and prospects for treatment. Nat. Rev. Neurol..

[3] Sanchez A.B., Kaul M. (2017). Neuronal Stress and Injury Caused by HIV-1, cART and Drug Abuse: Converging Contributions to HAND. Brain Sci..

[4] Jeevanjee S., Penko J., Guzman D., Miaskowski C., Bangsberg D.R., Kushel M.B. (2014). Opioid analgesic misuse is associated with incomplete antiretroviral adherence in a cohort of HIV-infected indigent adults in San Francisco. AIDS Behav..

[5] Merlin J.S., Tamhane A., Starrels J.L., Kertesz S., Saag M., Cropsey K. (2016). Factors Associated with Prescription of Opioids and Co-prescription of Sedating Medications in Individuals with HIV. AIDS Behav..

[6] Silverberg M.J., Ray G.T., Saunders K., Rutter C.M., Campbell C.I., Merrill J.O., Sullivan M.D., Banta-Green C.J., Von Korff M., Weisner C. (2012). Prescription long-term opioid use in HIV-infected patients. Clin. J. Pain.

[7] Chrousos G.P., Zapanti E.D. (2014). Hypothalamic-pituitary-adrenal axis in HIV infection and disease. Endocrinol. Metab. Clin. N. Am..

[8] Marik P.E., Kiminyo K., Zaloga G.P. (2002). Adrenal insufficiency in critically ill patients with human immunodeficiency virus. Crit. Care Med..

[9] Sharma N., Sharma L.K., Anand A., Gadpayle A.K., Gaurav K., Mukherjee S., Kulshreshtha B., Dutta D. (2018). Presence, patterns & predictors of hypocortisolism in patients with HIV infection in India. Indian J. Med. Res..

[10] Frye C.A., Paris J.J., Osborne D.M., Campbell J.C., Kippin T.E. (2011). Prenatal Stress Alters Progestogens to Mediate Susceptibility to Sex-Typical, Stress-Sensitive Disorders, such as Drug Abuse: A Review. Front. Psychiatry.

[11] Koob G.F., Volkow N.D. (2016). Neurobiology of addiction: A neurocircuitry analysis. Lancet Psychiatry.

[12] Kaul M., Zheng J., Okamoto S., Gendelman H.E., Lipton S.A. (2005). HIV-1 infection and AIDS: Consequences for the central nervous system. Cell Death Differ..

[13] Salahuddin M.F., Qrareya A.N., Mahdi F., Jackson D., Foster M., Vujanovic T., Box J.G., Paris J.J. (2020). Combined HIV-1 Tat and oxycodone activate the hypothalamic-pituitary-adrenal and -gonadal axes and promote psychomotor, affective, and cognitive dysfunction in female mice. Horm. Behav..

[14] Bokhari S.M., Yao H., Bethel-Brown C., Fuwang P., Williams R., Dhillon N.K., Hegde R., Kumar A., Buch S.J. (2009). Morphine enhances Tat-induced activation in murine microglia. J. Neurovirol..

[15] Gonek M., McLane V.D., Stevens D.L., Lippold K., Akbarali H.I., Knapp P.E., Dewey W.L., Hauser K.F., Paris J.J. (2018). CCR5 mediates HIV-1 Tat-induced neuroinflammation and influences morphine tolerance, dependence, and reward. Brain Behav. Immun..

[16] Fitting S., Zou S., Chen W., Vo P., Hauser K.F., Knapp P.E. (2010). Regional heterogeneity and diversity in cytokine and chemokine production by astroglia: Differential responses to HIV-1 Tat, gp120, and morphine revealed by multiplex analysis. J. Proteome Res..

[17] Pace T.W., Miller A.H. (2009). Cytokines and glucocorticoid receptor signaling. Relevance to major depression. Ann. N. Y. Acad. Sci..

[18] Goleva E., Kisich K.O., Leung D.Y. (2002). A role for STAT5 in the pathogenesis of IL-2-induced glucocorticoid resistance. J. Immunol..

[19] Pariante C.M., Pearce B.D., Pisell T.L., Sanchez C.I., Po C., Su C., Miller A.H. (1999). The proinflammatory cytokine, interleukin-1alpha, reduces glucocorticoid receptor translocation and function. Endocrinology.

[20] Raddatz D., Toth S., Schwörer H., Ramadori G. (2001). Glucocorticoid receptor signaling in the intestinal epithelial cell lines IEC-6 and Caco-2: Evidence of inhibition by interleukin-1beta. Int. J. Colorectal Dis..

[21] Bellavance M.A., Rivest S. (2014). The HPA—Immune Axis and the Immunomodulatory Actions of Glucocorticoids in the Brain. Front. Immunol..

[22] Franchimont D. (2004). Overview of the actions of glucocorticoids on the immune response: A good model to characterize new pathways of immunosuppression for new treatment strategies. Ann. N. Y. Acad. Sci..

[23] Leung D.Y., Martin R.J., Szefler S.J., Sher E.R., Ying S., Kay A.B., Hamid Q. (1995). Dysregulation of interleukin 4, interleukin 5, and interferon gamma gene expression in steroid-resistant asthma. J. Exp. Med..

[24] Baschant U., Tuckermann J. (2010). The role of the glucocorticoid receptor in inflammation and immunity. J. Steroid Biochem. Mol. Biol..

[25] Zapanti E., Terzidis K., Chrousos G. (2008). Dysfunction of the hypothalamic-pituitary-adrenal axis in HIV infection and disease. Hormones.

[26] Mukerji S.S., Misra V., Lorenz D.R., Chettimada S., Keller K., Letendre S., Ellis R.J., Morgello S., Parker R.A., Gabuzda D. (2020). Low Neuroactive Steroids Identifies a Biological Subtype of Depression in Adults with Human Immunodeficiency Virus on Suppressive Antiretroviral Therapy. J. Infect. Dis..

[27] Qiao S., Li X., Zilioli S., Chen Z., Deng H., Pan J., Guo W. (2017). Hair Measurements of Cortisol, DHEA, and DHEA to Cortisol Ratio as Biomarkers of Chronic Stress among People Living with HIV in China: Known-Group Validation. PLoS ONE.

[28] Christeff N., Gherbi N., Mammes O., Dalle M.T., Gharakhanian S., Lortholary O., Melchior J.C., Nunez E.A. (1997). Serum cortisol and DHEA concentrations during HIV infection. Psychoneuroendocrinology.

[29] Schürmeyer T.H., Müller V., von zurMühlen A., Schmidt R.E. (1997). Thyroid and adrenal function in HIV-infected outpatients. Eur. J. Med. Res..

[30] Norbiato G., Bevilacqua M., Vago T., Baldi G., Chebat E., Bertora P., Moroni M., Galli M., Oldenburg N. (1992). Cortisol resistance in acquired immunodeficiency syndrome. J. Clin. Endocrinol. Metab..

[31] Herman J.P., Adams D., Prewitt C. (1995). Regulatory changes in neuroendocrine stress-integrative circuitry produced by a variable stress paradigm. Neuroendocrinology.

[32] Kitraki E., Karandrea D., Kittas C. (1999). Long-lasting effects of stress on glucocorticoid receptor gene expression in the rat brain. Neuroendocrinology.

[33] Irusen E., Matthews J.G., Takahashi A., Barnes P.J., Chung K.F., Adcock I.M. (2002). p38 Mitogen-activated protein kinase-induced glucocorticoid receptor phosphorylation reduces its activity: Role in steroid-insensitive asthma. J. Allergy Clin. Immunol..

[34] Paris J.J., Liere P., Kim S., Mahdi F., Buchanan M.E., Nass S.R., Qrareya A.N., Salahuddin M.F., Pianos A., Fernandez N. (2020). Pregnane steroidogenesis is altered by HIV-1 Tat and morphine: Physiological allopregnanolone is protective against neurotoxic and psychomotor effects. Neurobiol. Stress.

[35] Nicolaides N.C., Galata Z., Kino T., Chrousos G.P., Charmandari E. (2010). The human glucocorticoid receptor: Molecular basis of biologic function. Steroids.

[36] Taniguchi Y., Iwasaki Y., Tsugita M., Nishiyama M., Taguchi T., Okazaki M., Nakayama S., Kambayashi M., Hashimoto K., Terada Y. (2010). Glucocorticoid receptor-beta and receptor-gamma exert dominant negative effect on gene repression but not on gene induction. Endocrinology.

[37] Webster J.C., Oakley R.H., Jewell C.M., Cidlowski J.A. (2001). Proinflammatory cytokines regulate human glucocorticoid receptor gene expression and lead to the accumulation of the dominant negative beta isoform: A mechanism for the generation of glucocorticoid resistance. Proc. Natl. Acad. Sci. USA.

[38] Bekhbat M., Mehta C.C., Kelly S.D., Vester A., Ofotokun I., Felger J., Wingood G., Anastos K., Gustafson D.R., Kassaye S. (2018). HIV and symptoms of depression are independently associated with impaired glucocorticoid signaling. Psychoneuroendocrinology.

[39] Bandaru V.V., Mielke M.M., Sacktor N., McArthur J.C., Grant I., Letendre S., Chang L., Wojna V., Pardo C., Calabresi P. (2013). A lipid storage-like disorder contributes to cognitive decline in HIV-infected subjects. Neurology.

[40] Cotto B., Natarajaseenivasan K., Ferrero K., Wesley L., Sayre M., Langford D. (2018). Cocaine and HIV-1 Tat disrupt cholesterol homeostasis in astrocytes: Implications for HIV-associated neurocognitive disorders in cocaine user patients. Glia.

[41] MohseniAhooyi T., Shekarabi M., Torkzaban B., Langford T.D., Burdo T.H., Gordon J., Datta P.K., Amini S., Khalili K. (2018). Dysregulation of Neuronal Cholesterol Homeostasis upon Exposure to HIV-1 Tat and Cocaine Revealed by RNA-Sequencing. Sci. Rep..

[42] Haughey N.J., Cutler R.G., Tamara A., McArthur J.C., Vargas D.L., Pardo C.A., Turchan J., Nath A., Mattson M.P. (2004). Perturbation of sphingolipid metabolism and ceramide production in HIV-dementia. Ann. Neurol..

[43] Fields J.A., Ellis R.J. (2019). HIV in the cART era and the mitochondrial: Immune interface in the CNS. Int. Rev. Neurobiol..

[44] Kesby J.P., Najera J.A., Romoli B., Fang Y., Basova L., Birmingham A., Marcondes M.C.G., Dulcis D., Semenova S. (2017). HIV-1 TAT protein enhances sensitization to methamphetamine by affecting dopaminergic function. Brain Behav. Immun..

[45] De Guglielmo G., Fu Y., Chen J., Larrosa E., Hoang I., Kawamura T., Lorrai I., Zorman B., Bryant J., George O. (2020). Increases in compulsivity, inflammation, and neural injury in HIV transgenic rats with escalated methamphetamine self-administration under extended-access conditions. Brain Res..

[46] McIntosh S., Sexton T., Pattison L.P., Childers S.R., Hemby S.E. (2015). Increased Sensitivity to Cocaine Self-Administration in HIV-1 Transgenic Rats is Associated with Changes in Striatal Dopamine Transporter Binding. J. Neuroimmune Pharmacol..

[47] Huynh Y.W., Thompson B.M., Larsen C.E., Buch S., Guo M.L., Bevins R.A., Murray J.E. (2020). Male HIV-1 transgenic rats show reduced cocaine-maintained lever-pressing compared to F344 wildtype rats despite similar baseline locomotion. J. Exp. Anal. Behav..

[48] Kesby J.P., Chang A., Najera J.A., Marcondes M.C.G., Semenova S. (2019). Brain Reward Function after Chronic and Binge Methamphetamine Regimens in Mice Expressing the HIV-1 TAT Protein. Curr. HIV Res..

[49] Wayman W.N., Chen L., Hu X.T., Napier T.C. (2016). HIV-1 Transgenic Rat Prefrontal Cortex Hyper-Excitability is Enhanced by Cocaine Self-Administration. Neuropsychopharmacology.

[50] Bali A., Randhawa P.K., Jaggi A.S. (2015). Stress and opioids: Role of opioids in modulating stress-related behavior and effect of stress on morphine conditioned place preference. Neurosci. Biobehav. Rev..

[51] Gaskill P.J., Miller D.R., Gamble-George J., Yano H., Khoshbouei H. (2017). HIV, Tat and dopamine transmission. Neurobiol. Dis..

[52] Raber J., Toggas S.M., Lee S., Bloom F.E., Epstein C.J., Mucke L. (1996). Central nervous system expression of HIV-1 Gp120 activates the hypothalamic-pituitary-adrenal axis: Evidence for involvement of NMDA receptors and nitric oxide synthase. Virology.

[53] Costa A., Nappi R.E., Polatti F., Poma A., Grossman A.B., Nappi G. (2000). Stimulating effect of HIV-1 coat protein gp120 on corticotropin-releasing hormone and arginine vasopressin in the rat hypothalamus: Involvement of nitric oxide. Exp. Neurol..

[54] Kino T., Gragerov A., Kopp J.B., Stauber R.H., Pavlakis G.N., Chrousos G.P. (1999). The HIV-1 virion-associated protein vpr is a coactivator of the human glucocorticoid receptor. J. Exp. Med..

[55] Sawaya B.E., Khalili K., Gordon J., Taube R., Amini S. (2000). Cooperative interaction between HIV-1 regulatory proteins Tat and Vpr modulates transcription of the viral genome. J. Biol. Chem..

[56] Kino T., Slobodskaya O., Pavlakis G.N., Chrousos G.P. (2002). Nuclear receptor coactivator p160 proteins enhance the HIV-1 long terminal repeat promoter by bridging promoter-bound factors and the Tat-P-TEFb complex. J. Biol. Chem..

[57] Bruce-Keller A.J., Turchan-Cholewo J., Smart E.J., Geurin T., Chauhan A., Reid R., Xu R., Nath A., Knapp P.E., Hauser K.F. (2008). Morphine causes rapid increases in glial activation and neuronal injury in the striatum of inducible HIV-1 Tat transgenic mice. Glia.

[58] Dong H., Wang S., Zeng Z., Li F., Montalvo-Ortiz J., Tucker C., Akhtar S., Shi J., Meltzer H.Y., Rice K.C. (2014). Effects of corticotrophin-releasing factor receptor 1 antagonists on amyloid-β and behavior in Tg2576 mice. Psychopharmacology.

[59] Yang X., Wang S., Rice K.C., Munro C.A., Wand G.S. (2008). Restraint stress and ethanol consumption in two mouse strains. Alcohol. Clin. Exp. Res..

[60] Hofford R.S., Prendergast M.A., Bardo M.T. (2015). Pharmacological manipulation of glucocorticoid receptors differentially affects cocaine self-administration in environmentally enriched and isolated rats. Behav. Brain Res..

[61] Lefevre E.M., Medley G.A., Reeks T., Alexander S., Burne T.H.J., Eyles D.W. (2017). Effect of the glucocorticoid receptor antagonist RU486 on MK-801 induced behavioural sensitisation. PLoS ONE.

[62] Leite L.M., Carvalho A.G., Ferreira P.L., Pessoa I.X., Gonçalves D.O., Lopes Ade A., Góes J.G., Alves V.C., Leal L.K., Brito G.A. (2011). Anti-inflammatory properties of doxycycline and minocycline in experimental models: An in vivo and in vitro comparative study. Inflammopharmacology.

[63] Paris J.J., Singh H.D., Ganno M.L., Jackson P., McLaughlin J.P. (2014). Anxiety-like behavior of mice produced by conditional central expression of the HIV-1 regulatory protein, Tat. Psychopharmacology.

[64] Fitting S., Scoggins K.L., Xu R., Dever S.M., Knapp P.E., Dewey W.L., Hauser K.F. (2012). Morphine efficacy is altered in conditional HIV-1 Tat transgenic mice. Eur. J. Pharmacol..

[65] Porsolt R.D., Bertin A., Jalfre M. (1977). Behavioral despair in mice: A primary screening test for antidepressants. Arch. Int. Pharmacodyn. Ther..

[66] Bourin M., Hascoët M. (2003). The mouse light/dark box test. Eur. J. Pharmacol..

